# Dynamics of Polyamines, Proline, and Ethylene Metabolism under Increasing Cold in Winter Oilseed Rape

**DOI:** 10.3390/ijms241411402

**Published:** 2023-07-13

**Authors:** Elžbieta Jankovska-Bortkevič, Sigita Jurkonienė, Virgilija Gavelienė, Vaidevutis Šveikauskas, Rima Mockevičiūtė, Irina Vaseva, Dessislava Todorova, Marija Žižytė-Eidetienė, Donatas Šneideris, Petras Prakas

**Affiliations:** 1 Nature Research Centre, Akademijos Str. 2, LT-08412 Vilnius, Lithuania; sigita.jurkoniene@gamtc.lt (S.J.); virgilija.gaveliene@gmail.com (V.G.); vaidevutis.sveikauskas@gamtc.lt (V.Š.); rima.mockeviciute@gamtc.lt (R.M.); marija.zizyte@gamtc.lt (M.Ž.-E.); donatas.sneideris@gamtc.lt (D.Š.); petras.prakas@gamtc.lt (P.P.); 2 Institute of Plant Physiology and Genetics, Bulgarian Academy of Sciences, Acad. G. Bonchev Str. Bl. 21, BG-1113 Sofia, Bulgaria; irina.vaseva@abv.bg (I.V.); dessita@bio21.bas.bg (D.T.)

**Keywords:** *Brassica napus*, cold stress, ethylene, oilseed rape, polyamines, proline, stress markers

## Abstract

Cold stress is among the most important environmental factors reducing the yield of crops. The present study aimed to investigate the impact of increasing cold stress conditions on winter oilseed rape polyamines, proline, and ethylene metabolism in acclimated and non-acclimated winter oilseed rape. This study was carried out under controlled conditions in the laboratory. The winter oilseed rape hybrid ‘Visby’ was used in the experiment. Acclimated and non-acclimated plants were subjected to a two-day-long increasing cold (from −1 °C to −3 °C) treatment. HPTLC, RT-qPCR, spectral analysis, and gas chromatography methods were used to analyse the levels of polyamines, gene expression, proline, and ethylene, respectively. This study showed a decrease in putrescine, spermidine, and spermine content during cold acclimation and a decrease in putrescine and spermidine levels at sub-zero temperatures. There were intensive changes in ADC2 gene expression, proline, and ethylene levels in non-acclimated plants: a substantial increase after exposure to −1 °C temperature and a sharp decrease after exposure to −3 °C temperature. The changes in these parameters were lower or absent in acclimated plants. The phenomena observed in this study add new insights to the knowledge about the plant stress response and suggest questions to be answered in the future.

## 1. Introduction

Adverse environmental factors affect the growth, yield potential, and distribution of plants. Thus, research on the plant stress mechanism and its control is one of the most important scientific fields to date. Physiological–biochemical reactions are activated in plants under stressful conditions. These are the defence mechanisms of plants, which help them to survive in unfavourable conditions when the level of an environmental factor exceeds the limit [1,2].

Oilseed rape (*Brassica napus* L.) is among the oldest crops grown in the world and is currently one of the most widely cultivated representatives of the *Brassicaceae* family. Its distribution mainly depends on climatic conditions [3,4]. Autumn–winter climate conditions in countries within the temperate climate zone encompass a wide range of natural phenomena. Research has shown that the combination and duration of their episodes determine the quality of winter oilseed rape wintering [5,6,7]. The main factors contributing to poor winter oilseed rape overwintering include a mild autumn, which makes non-acclimated (NA) plants susceptible to frosts, large daily temperature fluctuations, insufficient snow cover (especially snowless periods), long periods of cold resulting in drought, the formation of a thick ice crust during snowmelt, and others. Poor wintering may decline the yield of winter oilseed rape up to 90% or may result in total yield loss [8,9,10].

Different abiotic stresses are characterised by general and specific effects on plants. Current knowledge indicates that the plant response to low temperature consists of numerous components. It depends on the plant species, the stage of growth, the strength of the effect, the duration of action, the suddenness of temperature change, and interactions with other environmental factors. Nevertheless, many studies have been conducted to understand the mechanism underlying the plant response to cold, but there are still unknown aspects [1,9,10,11,12,13,14,15,16,17].

Polyamines (PAs) are organic polymeric compounds in all living organisms and are implicated in many biological functions. Studies have reported modified levels of PAs in plants under various stress conditions (temperature, drought, salt, heavy metals, etc.) [18,19]. Mutants of PA biosynthetic genes were shown to have a lower tolerance to abiotic stress, and transgenic plants with changed expression of PA biosynthetic genes including arginine decarboxylase (ADC), spermidine synthase (SPDS), and S-adenosylmethionine decarboxylase (SAMDC) were shown to have an increased tolerance to single and multiple stresses. PAs act as scavengers of oxygen radicals, serve as compatible solutes, stabilise macromolecules and cellular biomembranes under stress conditions, and participate in other stress-related plant responses. Thus, PAs are suggested to be used as markers for monitoring the impact of environmental stress on the metabolic status of plants [20,21,22].

Proline and ethylene have numerous functions and participate in the stress response of plants. PAs, proline, and ethylene are related through common precursors in their biosynthetic pathways. There is also evidence that catabolism of PAs contributes to proline accumulation. A positive correlation between proline accumulation and putrescine content and a negative correlation with spermidine content has been reported. Exogenous PA treatments were reported to increase the levels of PAs and proline. PAs have also been shown to regulate ethylene biosynthesis by inhibiting the accumulation of 1-aminocyclopropane-1-carboxylic acid (ACC). Nevertheless, there is some evidence of interaction, but the exact relationship between PAs, proline, and ethylene metabolism is still poorly understood. Many studies on the metabolism and interaction of these molecules have been conducted under drought, salinity, and other types of stress conditions. However, studies under cold stress are limited [23,24]. 

This study aimed to expand the scientific knowledge on the mechanism underlying the plant response to adverse environmental conditions by investigating the influence of increasing low-temperature stress on the metabolism of closely related plant stress biomarkers (PAs, proline, and ethylene) in winter oilseed rape. The insights obtained in this study under the specific environmental conditions may provide a clearer understanding of the plant response to cold and may be useful for future studies aiming to develop means for plant protection against abiotic stress.

## 2. Results

### 2.1. PA Content in the Leaves of Oilseed Rape Grown under Increasing Cold Conditions

**Figure 1:**
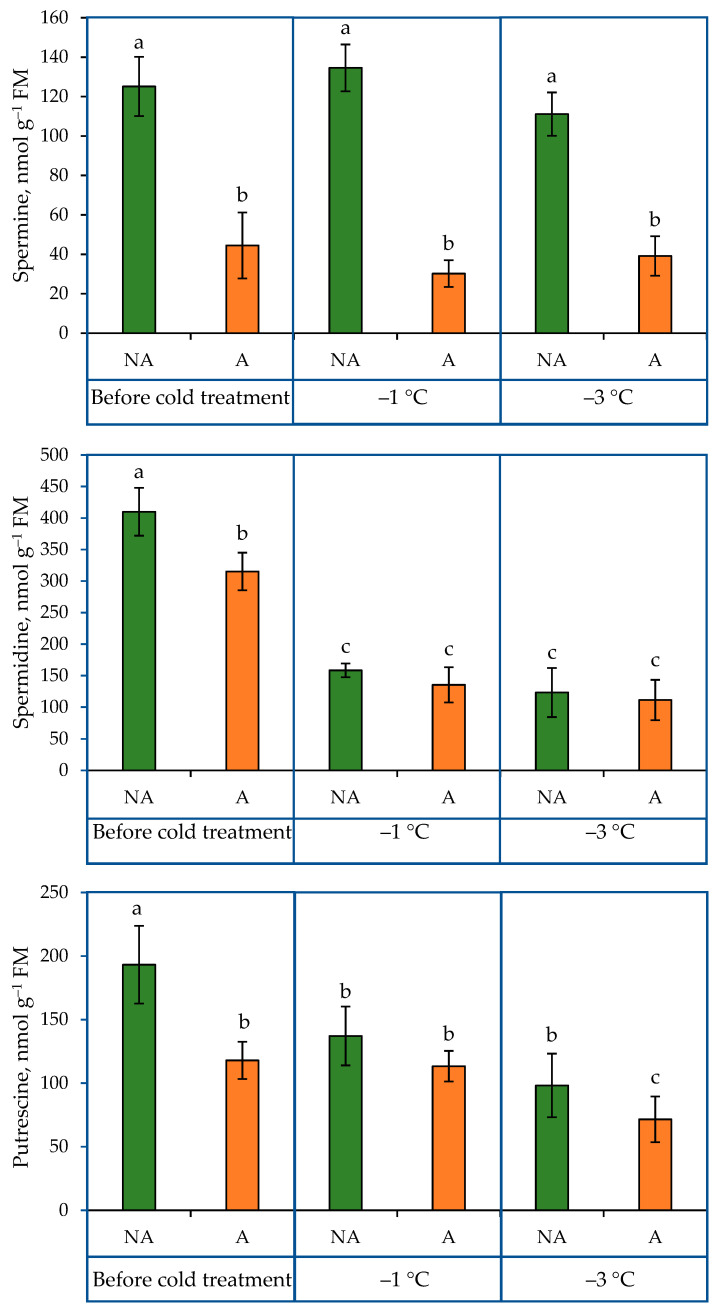
PA content in non-acclimated (NA) and acclimated (A) oilseed rape grown under increasing cold conditions. The bars represent the mean ± S.D. Different letters indicate statistically significant differences at the *p* < 0.05 level.

The PA content analysis showed that the acclimation treatment resulted in lower putrescine content values in oilseed rape plants (Figure 1). A significant decrease (29%) in putrescine content was observed in NA plants after the −1 °C treatment. A substantial drop in putrescine content in the acclimated (A) plants (by 42 nmol) was observed following the −3 °C treatment. Lower spermidine concentrations (by 23%) were observed in A plants compared to NA plants. The exposure of plants to the below-zero temperature treatment decreased and unified spermidine content both in NA and A plants. The spermine content was more than two-fold higher in NA than in the A oilseed rape plants before the cold treatment, and the subsequent exposure of plants to increasing cold conditions had no significant impact on the content of spermine in NA or A plants.

### 2.2. ADC2 Gene Expression Pattern in the Leaves of Oilseed Rape Grown under Increasing Cold Conditions

**Figure 2:**
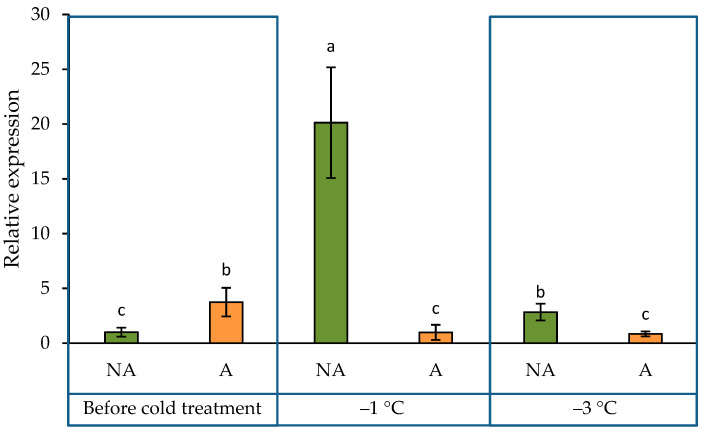
Gene expression pattern of arginine decarboxylase in non-acclimated (NA) and acclimated (A) oilseed rape grown under increasing cold conditions. The bars represent the mean ± S.D. Different letters indicate statistically significant differences at the *p* < 0.05 level.

The expression pattern of the PA biosynthetic gene ADC2 was analysed in this study. The acclimation treatment resulted in higher ADC2 expression levels in A than in NA oilseed rape plants (Figure 2). Interestingly, the −1 °C temperature treatment resulted in a completely opposite reaction in A and NA plants. It increased the expression of the ADC2 gene in NA plants (more than 10-fold) and decreased it in A plants. The subsequent exposure of plants to the −3 °C temperature treatment resulted in a significant drop in the expression pattern in NA plants but did not affect the expression pattern in A plants.

### 2.3. Proline Content in the Leaves of Oilseed Rape Grown under Increasing Cold Conditions

**Figure 3:**
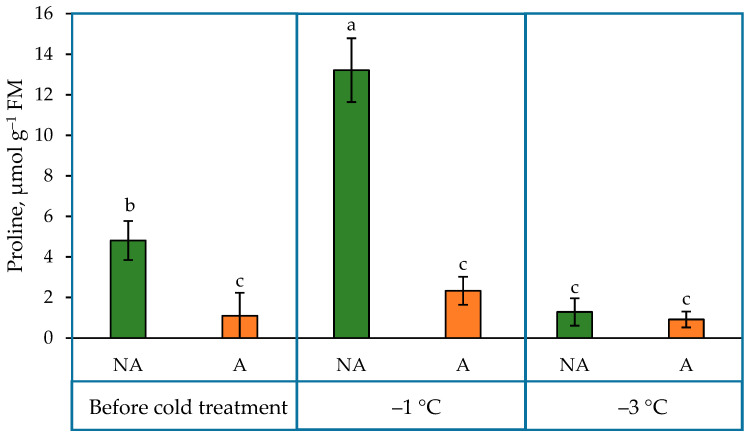
Proline content in non-acclimated (NA) and acclimated (A) oilseed rape grown under increasing cold conditions. The bars represent the mean ± S.D. Different letters indicate statistically significant differences at the *p* < 0.05 level.

Proline content was reduced by 3.72 µmol after the acclimation treatment (Figure 3) and did not change after the −1 °C and −3 °C temperature treatments. However, a significant rise (65%) in proline concentration was observed under the −1 °C temperature treatment, and the drop was also observed under the −3 °C treatment in NA oilseed rape plants.

### 2.4. Ethylene Emission in the Leaves of Oilseed Rape Grown under Increasing Cold Conditions

**Figure 4:**
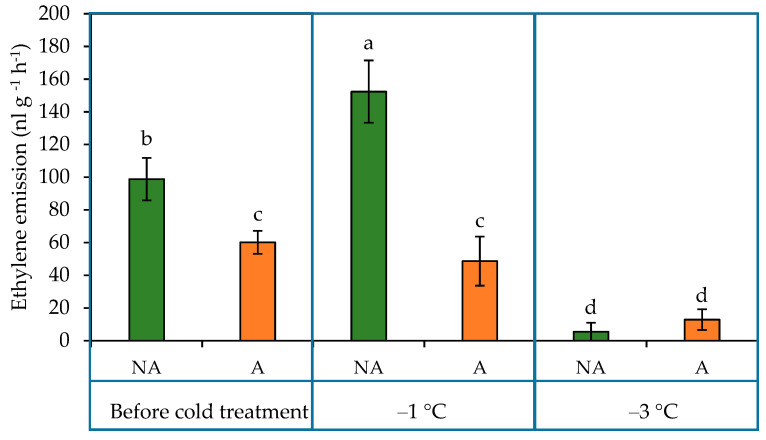
Ethylene emission from non-acclimated (NA) and acclimated (A) oilseed rape grown under increasing cold conditions. The bars represent the mean ± S.D. Different letters indicate statistically significant differences at the *p* < 0.05 level.

The levels of emitted ethylene (Figure 4) were 39% higher in NA than in A oilseed rape plants before treatment with sub-zero temperatures. A significant increase in ethylene emission was observed in N plants after the −1 °C temperature treatment. This temperature treatment did not affect the content of ethylene in A plants. Exposing plants to the −3 °C temperature treatment resulted in a significant ethylene emission drop both in NA and A plants.

## 3. Discussion

Temperature is one of the environmental factors that determine a plant’s geographical distribution. Temperatures above or below a specific range have a negative impact on the yield of agriculturally important crops and lead to economic loss. Some species from temperate regions, including winter oilseed rape, have developed an adaptive process named cold acclimation, which results in tolerance to low-non-freezing temperatures. Current knowledge indicates that plants initiate many physiological and metabolic responses under abiotic stress conditions, which are equipped with post-translational modifications and changes in gene expression. A deeper analysis of these responses may help develop plant protection methods against stress [22,25].

### 3.1. The Content of PAs

Many studies have revealed that the overall PA metabolism changes in response to many abiotic stresses. The changes in PA levels in response to stress are important in reducing the effects of stress. PAs were shown to be beneficial for protein homeostasis, detoxification of reactive oxygen species (ROS), activation of the antioxidative machinery, and molecular chaperone activity under stress conditions, etc. [26,27]. Earlier studies showed that PA levels were modified in various manners in different plant species and were dependent on the temperature level. An increase in the content of these secondary metabolites was the most commonly found response to abiotic stress, while a decrease was less frequently observed. According to the data from other studies, acclimation treatment increased the level of PAs in wheat, zucchini squash fruits, and winter wheat and decreased the levels in spring wheat [28,29]. The current study’s data showed that the acclimation treatment decreased the content of the analysed PAs in oilseed rape plants (Figure 1). The patterns in the changes in the levels of putrescine, spermidine, and spermine under increasing cold conditions were as follows: spermine levels did not change either in NA or A plants, and the levels of putrescine and spermidine decreased. Both the acclimation and increasing cold treatments impacted the content of PAs in the leaves of winter oilseed rape in this study. The results obtained in the current study support the idea that PAs are involved in plant responses to cold [30].

### 3.2. The Expression Pattern of PAs Biosynthetic Gene

The synthesis of putrescine from arginine by the ADC in plants is present under optimal conditions and various stresses (e.g., frosts, droughts, temperature changes, salinity, osmotic pressure, and other abiotic factors). It was shown that ADC1 and ADC2 are among the most significantly cold-induced genes in the PA biosynthesis pathway in plants [23,26]. Differential expression of ADC genes (ADC1 and ADC2) have been observed in *Arabidopsis* under various environmental stresses. ADC1 was activated mainly by cold, and ADC2 expression was induced with drought, high salinity, mechanical injury, and potassium. Another study showed that the level of both ADC1 and ADC2 transcripts increased during cold stress. Moreover, previous studies have shown that the up-regulation of ADC1 and ADC2 leads to an eventual accumulation of putrescine [21,27,31]. 

In this study, we analysed changes in the ADC2 gene expression pattern under acclimating and subzero conditions. The data showed that the ADC2 gene expression level was increased with the acclimation treatment (Figure 2). The −1 °C temperature treatment increased the expression of the ADC2 gene in non-acclimated plants (more than 10-fold) and decreased it in acclimated plants. The subsequent exposure of plants to the −3 °C temperature treatment resulted in a significant drop in the expression pattern in non-acclimated plants. However, it did not affect the expression pattern in acclimated plants. In the current study, we found that plant exposure to acclimation and increasing cold conditions changes the transcript level of ADC2 gene in winter oilseed rape.

### 3.3. The Content of Proline

Many studies have shown that plant exposure to suboptimal conditions alters proline metabolism and changes its content in the plant. This amino acid is known to act both as a primary and secondary metabolite. It is a compatible solute and contributes to low-temperature resistance and resistance to other stresses. Proline has been shown to enhance stress tolerance by improving membrane stability, ROS scavenging, and storage of energy and nitrogen [32,33,34,35].

What is more, proline was proposed to act as one of the osmolytes in enhancing plant stress tolerance [34,35]. However, in this study, we showed that the proline content significantly decreased during cold acclimation in winter oilseed rape ‘Visby’ plants (Figure 3). It should be noted that this disagrees with our previous studies, where proline content increased after acclimation treatment in two tested cultivars of winter oilseed rape [36]. Thus, other functions than osmoregulation could be suggested for proline in this case.

Many studies have shown an increase in proline accumulation in various plant species under stress conditions [33,35]. Our studies showed that the pattern of proline content under increasing cold treatment was different in A and NA plants. Intensive changes in proline metabolism were observed in NA plants under increasing cold conditions: a significant rise in proline concentration under the −1 °C temperature treatment and a drop in it under the −3 °C treatment in NA plants. However, no significant changes were observed in A plants.

### 3.4. The Level of Ethylene Emission

Ethylene is a gaseous plant hormone involved in various physiological and developmental processes as well as in different stress responses. The levels of this secondary metabolite and the expression of its signalling genes are rapidly upregulated in response to multiple abiotic stresses. Ethylene not only inhibits plant growth but also confers stress tolerance that maximises plant survival in adverse conditions by altering physiological and developmental activities in plants, e.g., it balances photosynthesis, controls stomatal aperture, cooperates with the ROS response pathway, promotes adventitious root formation, and regulates stem and petiole growth [22,24].

The ethylene biosynthesis pathway is related to one of the PA biosynthesis pathways. S-adenosylmethionine (SAM) formed from methionine is a common precursor of PAs and ethylene. Polyamines have been shown to regulate ethylene biosynthesis by inhibiting ACC accumulation. In addition, the common precursors sharing PAs and ethylene have antagonistic effects: PAs inhibit plant cell senescence, while ethylene promotes it [37,38,39].

The nature of the change in ethylene content varies depending on the species [22,40]. In the current study, levels of emitted ethylene (Figure 4) were higher in A than in NA oilseed rape plants before treatment with sub-zero temperatures. A significant increase in ethylene emission was observed in NA plants after the under −1 °C temperature treatment. This temperature treatment did not affect the content of ethylene in A plants. A subsequent exposure of plants to the −3 °C temperature treatment resulted in a significant drop in ethylene emission in both NA and A plants.

This study showed a strong increase in the ADC2 gene expression level, proline, and ethylene after exposure to the −1 °C temperature treatment and a sharp decrease after exposure to the −3 °C temperature treatment in N plants. The changes in these parameters were lower or absent in A plants. Also, a reduction in the amount of all PAs during cold acclimation and a decrease in spermidine and putrescine was observed at sub-zero temperatures. 

The phenomena observed in this study add new insights into the knowledge of the plant stress response and suggest questions to be answered in the future. For example, why did the total PA content decrease when oilseed rape plants were exposed to low temperatures? Do PAs serve as an energy source, or do they serve another purpose under low-temperature stress? How are the changes in ADC2 expression, proline, and ethylene levels related? The answers to these questions would add to the general understanding of the function and action of PAs, proline, and ethylene in plants and provide a prerequisite for the practical use of this knowledge in the future, e.g., to increase plant resistance to various stresses.

The specific environmental conditions designed for the present study widened the understanding of how the tested parameters operate. We believe that future studies on plant stress mechanisms in different plant species and under various specific environmental scenarios will be helpful in answering many fundamental questions and will provide new leads for crop protection against stress.

## 4. Materials and Methods

### 4.1. Plant Material and Growing Conditions

This study was carried out under controlled conditions in a laboratory. The winter oilseed rape hybrid ‘Visby’ (early, bred in Germany) was used in the experiment. Seeds were supplied by the Lithuanian Seeding Association. The seeds were sown in plastic cube pots of 10 cm edge length. Each pot contained nine plants. A mix (1:1 *v*/*v*) of garden compost and peat moss (pH 5.5–6.5) was used as the substrate. The substrate was irrigated using tap water. No fertilizer was used in this study.

**Figure 5:**
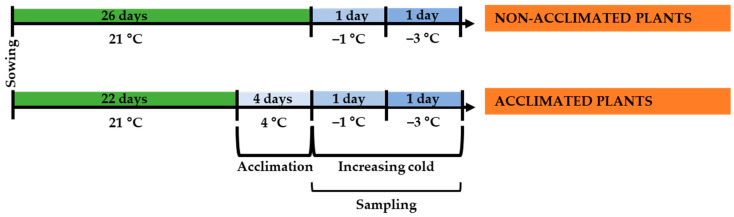
The design of this study.

Following the design of this study (Figure 5), plants were grown for 22 days under 21 ± 1 °C temperature (BBCH 13–14 stage, when 3–4 leaves were fully expanded) [41]. The photoperiod of 16/8 h day/night and 60 µmol m^−2^ s^−1^ cool white fluorescent light photon flux at the soil level in a conditioned growth chamber Climacell (MMM Medcenter Einrichtungen GmbH, Planegg, Germany) was set. The below-described experimental treatments were performed under the same illumination. 

### 4.2. Low-Temperature Treatments

The low-temperature treatments were performed in Friocell (MMM Medcenter Einrichtungen GmbH, Planegg, Germany) cooling incubators. For the 4-day-long cold acclimation treatment (Figure 5), the pots with 22-day-old plants were divided into two subgroups. One subgroup was treated under 21 ± 1 °C temperature (non-acclimated, NA plants), and another subgroup was treated under 4 ± 1 °C temperature (acclimated, A plants).

Next, for the two-day-long increasing cold (from −1 to −3 °C) treatment (Figure 5), on the first day, the temperature was gradually lowered by 2 °C h^−1^ until −1 °C was reached. On the second day of the treatment, the temperature was reduced by the same rate to −3 °C.

Fully expanded leaf blades were collected for assays after the cold acclimation and each day of the increasing cold treatment. Freshly harvested samples were used for ethylene emission analysis. Samples collected for PA content, gene expression, and proline analyses were placed in liquid nitrogen and stored in an ultra-low freezer (Skadi Green line, Taiwan, China) at −80 °C.

### 4.3. Determination of PA Content

The content of PAs was determined according to the method reported by Madhubala [42] with minor modifications. Frozen samples (0.5 g) were homogenized in 2 mL of 2% (*v*/*v*) perchloric acid in chilled mortars (4 °C). The residue was removed with centrifugation at 13,800× *g* for 15 min. Next, 600 μL of the supernatant was used for the derivatisation reaction. Dansyl chloride (5 mg/mL) was dissolved in acetone. Then, 2× dansyl chloride (1200 μL) and 1× sodium carbonate (600 μL) were added to the samples and then vortexed and incubated in the dark for 16 h. The excess dansyl chloride was removed by adding 300 μL of proline (Roth) solution in deionised water (150 mg/mL) and stored in the dark for a further 30 min. Next, 1500μL of toluene was used to extract dansyl amides from the solution. After vortexing and centrifugation for 10 min at 13,800× *g*, the dansyl amides in toluene were further analysed with HPTLC using CAMAG (Switzerland) equipment and the visionCATS 2.5 ver. (Switzerland) programme. The automatic TLC Sampler 4 was used to apply standards and samples as 6 mm bands on 10 cm × 20 cm glass silica gel SIL G-25 UV_254_ chromatography plates. Standard solutions (1 M) of putrescine, spermidine, and spermine (Sigma-Aldrich, St. Louis, MO, USA) were prepared by dissolving them in deionised water. The elution was carried out in the Automatic Developing Chamber 2. A 25:4 ratio of chloroform:triethylamine mobile phase was used. The dried plate was transferred to the TLC visualiser 2 and illuminated at 365 nm. Finally, the absorption was measured using the TLC Scanner 4. The obtained data were derived from the results of sample peaks, analogous to ascorbic acid in the calibration curve. The content of PAs was determined according to the standard curve of known PA concentrations.

### 4.4. RT-qPCR

Total RNA from frozen leaf tissues weighing 150 mg was isolated and purified using the commercial RNA isolation kit: Quick-RNA Plant MiniPrep™ Kit (ZymoResearch, Irvine, CA, USA). DNase I set (ZymoResearch, Irvine, CA, USA) was applied to avoid genomic DNA contamination. The quantity and quality of the isolated RNA were evaluated using a NanoPhotometer® P330 spectrophotometer (Implen, Westlake Village, CA, USA). RNA purity was assessed using the ratio of optical density (OT) absorbance OT 260 nm/OT 280 nm (range 1.80 to 2.05) and OT 260 nm/OT 230 nm (range 2.00 to 2.30). RNA integrity was assessed using electrophoresis in a 1% agarose gel stained with ethidium bromide. RNA samples were stored at −20 °C until the RT-qPCR analysis.

Complementary DNA (cDNA) was synthesised in a PCR thermocycler Lab Cycler (SensoQuest, Göttingen, Germany) using the commercial Verso cDNA Synthesis Kit (Thermo Scientific™, Waltham, MA, USA). Under the different experimental conditions, two commonly used reference genes (HTB1 and UBC21) (Table 1) [43,44,45] were used for gene expression analysis in current study.

The gene encoding arginine decarboxylase 2 (ADC2) was used as the target gene for this study. Primers for the target gene were designed using the Primer3Plus programme, choosing the optimal length of the nucleotide sequence in the intronic part of the gene (Table 2).

The qRT-PCR analysis of the samples was performed with the Azure Cielo (USA) real-time PCR system using the commercial Maxima SYBR Green qPCR Master Mix (2X) kit (Thermo Scientific™, Waltham, MA, USA) according to the manufacturer’s recommendations in three biological and technical replicates. Data were processed with Quant Studio™ Design and Analysis Software version v1.5.2 (Thermo Scientific™, Waltham, MA, USA). Gene expression levels were calculated using the delta–delta Ct method according to the formula:

Gene expression level = 2^−∆∆Ct^, where ∆∆Ct = ∆Ct (affected sample) − ∆Ct (unaffected sample); ∆Ct = Ct (target gene) − Ct (reference gene) [43,46].

### 4.5. Proline Content Analysis

A colour reaction of acidified ninhydrin [47] was used to determine proline content. In brief, 0.5 g of the plant material was ground for 2 min in a chilled mortar and pestle with 10 mL of 3% sulphosalicylic acid (Roth, Germany) and extracted for 14 h at 4 °C. Extracts were centrifuged at 700 g (centrifuge MPW–351 R, Poland) for 20 min. Ninhydrin (1.25 g) (Roth, Germany) was dissolved in glacial acetic acid (30 mL) (Roth) and 6 M phosphoric acid (20 mL) (SIGMA-ALDRICH Chemie GmbH, Schnelldorf, Germany) using warming and shaking until dissolved to prepare acidified ninhydrin. The mixture of supernatant, acetic acid, and acidified ninhydrin was heated for 1 h at 100 °C in a heater BLOCKTHERMOSTAT BT 200 (Kleinfeld Labortechnik, Gehrden, Germany). Hot samples were cooled in an ice bath for 15 min. Then, toluene (Roth, Germany) was added to extract the chromophore. The mixture was vigorously shaken and incubated in the dark for 1 h. The absorbance was analysed at 520 nm using a multi-sample quartz cuvette (Hellma, Müllheim im Markgräflerland, Germany) and Rainbow microplate reader (SLT Labinstruments, Grödig, Austria). Toluene was used for the blank. The content of proline was calculated using the curve of known L-proline concentrations. Calculations were carried out using the SLT programme (SLT Labinstruments, Grödig, Austria). Results were estimated as μmol of proline per gram of fresh mass. 

### 4.6. Ethylene Emission Analysis

Ethylene emission from leaves was evaluated using the method of Child et al. [48] with slight modifications. Samples were weighed, placed in 40 mL volume glass vials, and sealed with PTFE/Si septa (Agilent Technologies, Santa Clara, CA, USA ) followed by 24 h of incubation at 21 ± 1 °C in the dark. Then, 1 mL of sample gas was injected using a gas-tight syringe (Agilent Technologies, Santa Clara, CA, USA) into a gas chromatograph (Thermo Scientific* FOCUS GC, Waltham, MA, USA). A stainless-steel column (matrix 80/100 Thermo Scientific* PROPAC R, Waltham, MA, USA) and a hydrogen flame ionization detector were used in the analysis. The temperatures of 110, 90, and 150 °C were set for the injector, column, and detector, respectively. The carrier gas was helium (AGA, Vilnius, Lithuania). Ethylene (Messer, Bad Soden, Germany) was used as a standard for calibration. The results were expressed as nanolitres of ethylene per gram of fresh mass per hour.

### 4.7. Statistics

The statistical analyses were performed using the SPSS Statistics 23 (IBM, Armonk, NY, USA) program. The research data were subjected to an analysis of variance (ANOVA). Differences at *p* < 0.05 were considered to be significant. Multiple comparisons for mean values were performed using a post hoc test. The values were presented as mean ± standard deviation (SD). 

## Figures and Tables

**Table 1:** Reference genes used in the study.

Nr.	Gene Name	Amplicon Size, bp	Primer Sequence	Oligomer Name	T_m_, °C
1.	Histone superfamily protein	142	TATTCGAGAAGCTCGCCCAG	HTB1-F	62
			TTGGTTCCTTCAGAGACGGC	HTB1-R	62
2.	Ubiquitin-conjugating enzyme 21	176	TATCCTCTGCAGCCTCCTCA	UBC21-F	62
			CTGTCTGCCTCAGGATGAGC	UBC21-R	64

**Table 2:** Target gene data.

Gene Name	Amplicon Size, bp	Primer Sequence	Oligomer Name	T_m_, °C
Arginine decarboxylase	240	TTGTATGCTTGACTGTCTCCAG	BnADC2Pe-F	64
		ACAGCTTCAGCGTACTCCTC	BnADC2Pe-R	62

## Data Availability

All data are provided herein.

## Abbreviations

A	acclimated
NA	non-acclimated
PAs	polyamines
ADC	arginine decarboxylase
SPDS	spermidine synthase
SAMDC	S-adenosylmethionine decarboxylase
ROS	reactive oxygen species
SAM	S-adenosylmethionine
HTB	histone superfamily protein
UBC	ubiquitin-conjugating enzyme
